# Genome-Wide Transcriptomic Analysis Reveals a Regulatory Network of Oxidative Stress-Induced Flowering Signals Produced in Litchi Leaves

**DOI:** 10.3390/genes11030324

**Published:** 2020-03-18

**Authors:** Xingyu Lu, Sheng Yu, Peitao Lü, Houbin Chen, Silin Zhong, Biyan Zhou

**Affiliations:** 1 Guangdong Litchi Engineering Research Center, College of Horticulture, South China Agricultural University, Guangzhou 510642, China; luxingyu90@163.com (X.L.); hbchen@scau.edu.cn (H.C.); 2 State Key Laboratory of Agrobiotechnology, School of Life Sciences, Chinese University of Hong Kong, Hong Kong, China; yusheng@link.cuhk.edu.hk (S.Y.); ptlv@cau.edu.cn (P.L.)

**Keywords:** flowering, reactive oxygen species, induction, transcriptome, litchi

## Abstract

Litchi is an important subtropical fruit tree that requires an appropriately low temperature to trigger floral initiation. Our previous studies have shown that reactive oxygen species (ROS) are involved in litchi flowering. To identify oxidative stress-induced flowering related genes in leaves, ‘Nuomici’ potted trees were grown at medium low-temperature conditions (18/13 °C for day/night, medium-temperature). The trees were treated with the ROS generator methyl viologen dichloride hydrate (MV) as the MV-generated ROS treatment (MM, medium-temperature plus MV) and water as the control treatment (M, medium-temperature plus water). Sixteen RNA-sequencing libraries were constructed, and each library generated more than 5,000,000 clean reads. A total of 517 differentially expressed genes (DEGs) were obtained. Among those DEGs, plant hormone biosynthesis and signal transduction genes, ROS-specific transcription factors, such as AP2/ERF and WRKY genes, stress response genes, and flowering-related genes *FLOWERING LOCUS T1* (*FT1*) and *FLOWERING LOCUS T2* (*FT2*) were significantly enriched. Then, as a confirmatory experiment, the potted trees were uniformly sprayed with MV, N,N’-dimethylthiourea (DMTU, ROS scavenger) plus MV, and water at medium-temperature. The results showed that the MV-generated ROS promoted flowering and changed related gene expression, but these effects were repressed by DMTU treatment. The results of our studies indicate that ROS could promote flowering and partly bypass chilling for litchi flowering.

## 1. Introduction

Litchi is an evergreen fruit tree commercially cultivated in southern Asia, South Africa, and Australia. Floral initiation in litchi is triggered by low temperatures and enhanced by drought in autumn and winter [1,2,3]. Litchi trees grown in controlled growth chambers with stably low temperature can produce large and high-quality panicles. Interestingly, litchi trees grown under high-temperature conditions with only a few leaves treated with low temperature could still produce flowers, although the panicles were small [4], suggesting that these low-temperature-treated leaves can produce flowering signals that could be transported to the apical meristem to activate flowering through the phloem. In the model plant Arabidopsis, CONSTANS (CO) is regarded as a direct activator of FLOWERING LOCUS T (FT), and the FT protein is indicated to be a long-seeking florigen that migrated from leaves to the shoot apical meristem (SAM) to promote floral initiation [5,6]. In other plants such as cucurbit and rice, the FT protein or its analog is proven to be a long-distance florigenic signal transported in the phloem to the SAMs and to activate the transition from the vegetative meristem to the inflorescence meristem [7,8]. Hence, two kinds of tissues may play important roles in flowering. The leaves may act as sites for signal production, while SAMs may act as sites for the signal reaction. In our previous studies, we focused on the sites for the signal reaction of litchi flowering, the SAMs. 

We have previously shown that reactive oxygen species (ROS) promoted flowering and decreased the chilling requirement for flowering [9] and obtained a few ROS-responsive Expressed sequence tag (EST) clones [10]. We also performed RNA-sequencing (RNA-Seq) to obtain a global transcriptome of the apical meristem, and to identify genes involved in the transformation from vegetative meristems to inflorescence meristems triggered by ROS and chilling [11]. In the present study, we aimed to identify genes involved in leaf-generated signals for ROS-promoted flowering in litchi. We used a controlled experimental system in which temperatures were accurately controlled. In this system, ‘Nuomici’ potted trees were grown at medium low-temperature conditions (18/13 °C for day/night, medium-temperature) and were treated with an ROS generator, methyl viologen dichloride hydrate (MV) as MV-generated ROS treatment (MM, medium-temperature plus MV) and water as controls (M, medium-temperature plus water). Then, 16 RNA-Seq libraries from leaves were constructed and sequenced. From these RNA-Seq data, we identified genes potentially involved in ROS-promoted flowering. By these studies, we identified potential flowering-related genes expressed in leaves that bypass chilling and provide essential information for genetic regulation of litchi flowering.

## 2. Materials and Methods

### 2.1. Plant Material and Experiment Procedures

Five-year-old air-laying potted trees (*Litchi chinenesis* cv. Nuomici) were used in this study. Twenty potted trees (1–1.5 m height) with leaves of the terminal shoots at the same maturity stage were selected. All trees were cultivated in the experimental orchard of South China Agricultural University, Guangzhou, China (lat. 23°9040” N, long. 113°21018” E) and were grown in 30 L pots with loam, mushroom cinder and coconut chaff (v: v: v, 3:1:1). Trees were transferred to a growth chamber at 18 °C/13 °C (day/night temperature, 12 h day and 12 h night) with a relative humidity of 75%–85% and natural light. Ten trees were sprayed with 60 µM MV according to our previous studies [9] as a treatment of medium-temperature plus MV (MM), whereas the remaining 10 trees were sprayed with water as a medium-temperature control (M). Trees were rewarmed to 25 °C/20 °C after 63 days of treatment. After rewarming for 15 d when panicle primordia appeared, the percentage of flowering trees was calculated. Leaves at the time points of 3 d, 33 d, 63 d, and 78 d were sampled. Leaves of the terminal shoots from 5 trees were pooled together as a replicate, and those from another 5 trees were pooled as another replicate. All the samples were immediately frozen in liquid nitrogen and stored at −80°C for RNA extraction.

To further confirm the role of ROS in flowering promotion in litchi, fifteen six-year-old air-laying potted ‘Nuomici’ trees with mature terminal shoots were used. Treatments were also performed at a growth chamber at 18 °C/13 °C (day/night temperature, 12 h day and 12 h night). Five replicated trees were sprayed with 40 µM MV as the medium-temperature plus MV treatment (MM). The other five trees were initially sprayed with 2 mM of N,N’-dimethylthiourea (DMTU) and then sprayed with 40 µM MV 12 h later as the medium-temperature plus DMTU and MV treatment (MMD). The remaining five trees were sprayed with water as the medium-temperature control (M). All the dosages for the chemicals were in accordance with our previous studies [9]. Trees were rewarmed to 25 °C/20 °C after 63 days of treatment. After rewarming for 20 d when panicle primordia appeared, the percentage of flowering trees was calculated. Leaves of the three treatments at the time points of 3 d, 33 d, 63 d, and 83 d were sampled. All the samples were immediately frozen in liquid nitrogen and stored at −80°C for RNA extraction.

### 2.2. RNA Isolation, Library Construction and Sequencing

Total RNA was extracted using the RNAprep Pure Plant Kit (Polysaccharides and Polyphenolics-rich) (Tiangen Biotech, Beijing, China). For RNA-Seq library construction, 5 μg of total RNA with an appropriate amount of Oligo-dT25 beads (Invitrogen, Carlsbad, CA, USA) was used. The mRNA was fragmented into short fragments and reverse transcribed into cDNA by random primers. Second-strand cDNA was synthesized using DNA polymerase I, RNase H and dNTPs. After that step, the cDNA fragments were purified by Agencourt AMPure XP (Beckman Coulter, Pasadena, CA, USA). The purified cDNA fragments were end-repaired, modified with poly (A) tails, and ligated to Illumina sequencing adapters. The size-selected fragments were amplified and purified. Sixteen libraries were sequenced withIllumina HiSeq™ 2500 (BGI Co. Ltd., Shenzhen, China). The raw sequence data reported in this paper have been deposited in the Genome Sequence Archive [12] in the Beijing Institute of Genomics (BIG) Data Center [13], Beijing Institute of Genomics, Chinese Academy of Sciences, under accession numbers CRA001770 and are publicly accessible at https://bigd.big.ac.cn/gsa.

### 2.3. RNA-Seq Data Analysis

Adapters were cut from raw reads, and the low-quality sequences were removed. rRNA sequences were filtered against ribosomal RNA using Bowtie2 (version 2.1.0). Then, cleaned reads were mapped to the litchi genome using HISAT2 (v2.1.0). The read counts and fragments per kilobase of transcript per million mapped reads (FPKM) of each gene were calculated using eXpress (v1.5.1, https://pachterlab.github.io/eXpress/index.html). Differentially expressed genes (DEGs) were determined using OmicShare tools (http://www.omicshare.com/tools/Home/Soft/diffanalysis). The R package is edgeR (http://www.bioconductor.org/packages/release/bioc/html/edgeR.html), and significant DEGs were restricted with false discovery rate (FDR) ≤ 0.05 and the absolute value of fold-change ≥ 2.

### 2.4. Quantitative Real-Time PCR Analysis

Total RNA was extracted using the RNAprep Pure Plant Kit (Polysaccharides and Polyphenolics-rich) (Tiangen Biotech). First-strand cDNA was synthesized from 1 μg of extracted total RNA using TransScript One-Step gDNA Removal and cDNA Synthesis SuperMix (Transgen Biotech). Quantitative real-time RT-PCR (qRT-PCR) primers F/R (Table S1) were designed using Primer 5.0 software and synthesized at Sangon Co. Ltd. (Shanghai, China). The litchi homolog β-actin (GenBank accession number: HQ588865.1) was used as the reference gene. qRT-PCR was performed on a CFX96 real-time PCR machine (Bio-Rad, Hercules, CA, USA) with 2× RealStar Green Power Mixture (GenStar BioSolutions, Beijing, China). qPCR was performed at 95 °C for 10 min followed by 40 cycles of 95 °C for 15 s, 60 °C for 30 s, and 72 °C for 30 s in 96-well optical reaction plates. Each qRT-PCR analysis was performed in triplicate. The transcript quantification of the genes was performed in relation to *Actin*, and they were calculated using the 2^−△△CT^ method [14].

### 2.5. Construction of Gene Co-expression Networks

According to the classification described by You et al. and Sudawan et al. [15,16], DEGs involved in ROS signaling, stress response, and flowering were collected to establish a gene co-expression network. The weighted gene co-expression network analysis (WGCNA, v1.48) package in R was chosen to obtain the weight value of those selected genes, and Cytoscape (version 3.2.1) was used to analyze the intergenic connectivity and construct the network. A weight value > 0.15 was subjected as the threshold value, the connecting lines (edges) of color from light yellow to pink correspond to the weight value, and deeper colors represent higher values. The size of the node corresponds to the connectivity analyzed by NetworkAnalyzer of Cytoscape associated with the degrees (number of the edges) and weight value.

### 2.6. Statistical Analysis

Data were analyzed by SPSS (version 19.0; IBM Corp., Armonk, NY, USA) using Student’s *t*-test and Duncan’s multiple range test. The expression of candidate genes was presented as a heat map diagram using the pheatmap package (http://www.r-project.org/) or a line chart using Origin (version 9.1; OriginLab Corp., Northampton, MA, USA).

## 3. Results

### 3.1. MV-Generated ROS Promote Flowering

**Figure 1:**
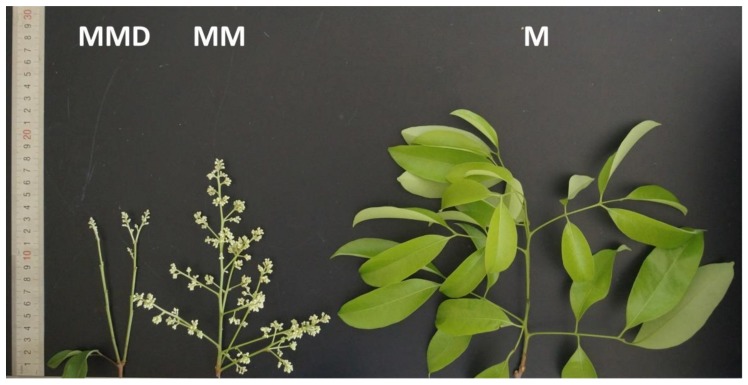
Morphology of the panicles or new flushes after medium-temperature treatment. ‘Nuomici’ trees were transferred to a growth chamber at 18 °C/13 °C (day/night temperature, 12 h day and 12 h night) as medium-temperature treatment. The trees were sprayed with 40 µM methyl viologen dichloride hydrate (MV) as the medium-temperature plus MV treatment (MM), 2 mM N,N’-dimethylthiourea (DMTU) in combination with 40 µM MV as the medium-temperature plus DMTU and MV treatment (MMD), and water as the medium-temperature control (M). MMD shows a small panicle in the MMD-treated trees. MM shows a large panicle in the MM-treated trees. M shows a new flush in the M-treated trees.

In our previous study, we found that after a period of low-temperature (LT, 15 °C/8 °C) treatment, ‘Nuomici’ litchi trees were induced to flower in 100% of cases, whereas under medium-temperature (MT, 18 °C/13 °C) conditions, litchi trees produced poor flowers in 20% of cases. However, when trees at medium-temperature were sprayed with an ROS donor MV, they were induced to flower in 80% of the trees [11]. To further confirm the promoting effects of ROS on flowering under medium-temperature conditions, we conducted another experiment using the ROS scavenger N,N’-dimethylthiourea (DMTU). As shown in Figure 1 and Table 1, the MV-generated ROS (MM)-treated trees could produced large panicles measuring 12.6 cm in length and 5.7 cm in width. In contrast, trees without ROS treatment (M) produced no flowers with new flushes, whereas trees treated with ROS in combination with the ROS scavenger DMTU (MMD) produced considerably fewer flowers, although the percentage of flowering trees was the same as with MM. The results indicated that ROS promoted flowering under medium-temperature conditions, but this effect could be reduced by the ROS scavenger DMTU.

### 3.2. Digital Transcriptomic Analysis

To identify genes potentially related to oxidative stress-induced flowering signals in leaves, we collected leaves from the ROS-treated and water-treated trees at 3 d, 33 d, 63 d, and 78 d of medium-temperature treatment. We constructed 16 RNA-Seq libraries for the four time points for the MM-treated and M-treated leaves. As shown in Table 2, we generated 5.17–12.15 × 10^6^ raw reads and obtained 4.95–11.55 × 10^6^ clean reads from the libraries with clean read ratios of more than 88%. We then mapped the clean reads to the litchi genome data, and the alignment rates were all above 85%.

### 3.3. Identification of Differentially Expressed Genes

**Figure 2:**
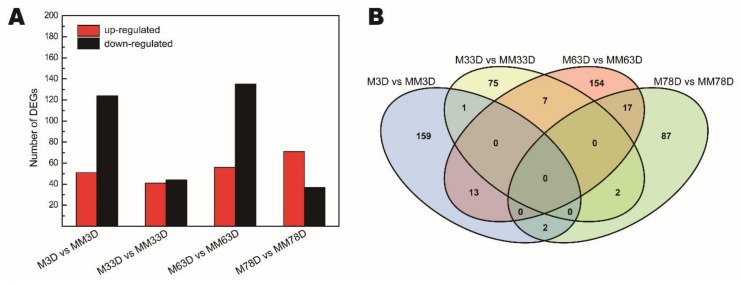
Differentially expressed genes (**A**) and Venn diagram of the DEGs (**B**) in response to ROS. ‘Nuomici’ litchi trees were transferred to a growth chamber at 18 °C/13 °C (day/night temperature, 12 h day and 12 h night) as medium-temperature treatment. The trees were sprayed with MV as the medium-temperature plus MV treatment (MM) and water as the medium-temperature control (M). M3D, M33D, M63D, and M78D indicate 3 d, 33 d, 63 d, 78 d of water treatment, respectively. MM3D, MM33D, MM63D, and MM78D respectively, indicate 3 d, 33 d, 63 d, 78 d of MV treatment under medium-temperature conditions.

We remapped the unique match reads from the 16 libraries to the litchi genome data and normalized the unigene reads to FPKM values. Correlation analysis of the replicates of the two treatments (M and MM) and four time points (3 d, 33 d, 63 d, and 78 d) showed high repeatability of the sequencing samples (Figure S1). Then we conducted a pair comparison of M and MM at the same time point to identify differentially expressed genes (DEGs) using edgeR. As shown in Figure 2A and Table S2, 517 DEGs were obtained, during which 175 DEGs were from the 3 d time point, and 358 DEGs were from the later time points, including the 33 d, 63 d, and 78 d time points. At the 3 d, 33 d, and 63 d time points, more down-regulated DEGs (from M to MM) than the up-regulated DEGs were found. At the 78 d time point, more up-regulated DEGs were found than the down-regulated DEGs (Figure 2A). The DEGs between the two time points seemed specific. For example, only one DEG was found from M3D vs. MM3D and M33D vs. MM33D (Figure 2B). 

### 3.4. Identification of Genes Potentially Involved in Oxidative Stress-Induced Flowering

**Figure 3:**
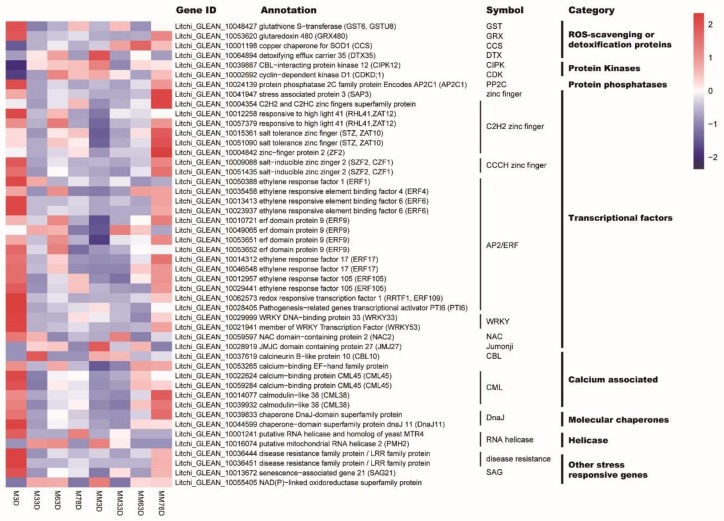
Heat map diagram showing the expression profiles of DEGs identified from the 3 d time point. ‘Nuomici’ litchi trees were transferred to a growth chamber at 18 °C/13 °C (day/night temperature, 12 h day and 12 h night) as medium-temperature treatment. The trees were sprayed with MV as the medium-temperature plus MV treatment (MM) and water as the medium-temperature control (M). M3D, M33D, M63D, and M78D indicate 3 d, 33 d, 63 d, 78 of water treatment, respectively. MM3D, MM33D, MM63D, and MM78D respectively, indicate 3 d, 33 d, 63 d, 78 d of MV treatment under medium-temperature conditions. Fragments per kilobase of transcript per million mapped reads (FPKM) values were normalized to Z-score.

**Figure 4:**
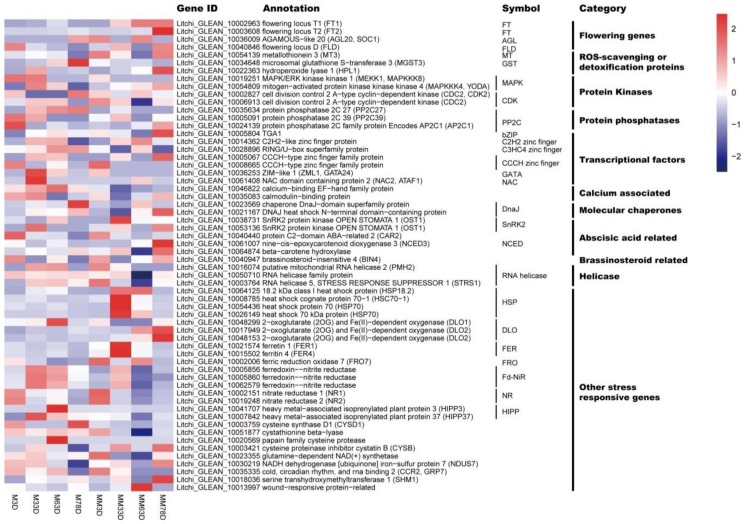
Heat map diagram showing the expression profiles of DEGs identified from the 33 d, 63 d, or 78 d time points. ‘Nuomici’ litchi trees were transferred to a growth chamber at 18 °C/13 °C (day/night temperature, 12 h day and 12 h night) as medium-temperature treatment. The trees were sprayed with MV as the medium-temperature plus MV treatment (MM) and water as the medium-temperature control (M). M3D, M33D, M63D, and M78D indicate 3 d, 33 d, 63 d, 78 of water treatment, respectively. MM3D, MM33D, MM63D, and MM78D respectively, indicate 3 d, 33 d, 63 d, 78 d of MV treatment under medium-temperature condition. FPKM values were normalized to Z-score.

From the 517 DEGs, we identified those related to ROS signaling, stress response, and flowering-related genes. As a result, we found 48 DEGs at the 3 d time point, representing the early ROS responsive genes, and 60 DEGs at the 33, 63, and 78 d time points, representing the late ROS responsive genes. The DEGs were classified into 11 groups. These genes were ROS-scavenging-related, abscisic acid-related, brassinosteroid-related, calcium-associated, flowering-related, and other stress-responsive genes, as well as helicase, molecular chaperone, protein phosphatase, protein kinase, and transcriptional factor encoding genes (Figure 3 and Figure 4). Interestingly, at the 3 d time point, many more transcription factors encoding DEGs were found compared to those at the later time points. Twenty-seven transcription factor encoding DEGs out of the total 48 DEGs were identified at the 3 d time point, while only seven of those out of the 60 DEGs were found at the later time points. Moreover, flowering-related DEGs, including *FT1* (Litchi_GLEAN_10002963), *FT2* (Litchi_GLEAN_10003608), *SUPPRESSOR OF OVEREXPRESSION OF CO1* (*SOC1*, Litchi_GLEAN_10036009), and *FLOWERING LOCUS D* (*FLD*, Litchi_GLEAN_10040846), were only found at the later time points. *AP2C1* (Litchi_GLEAN_10024139), a protein phosphatase-encoding gene, and *putative mitochondrial RNA helicase 2* (*PMH2*, Litchi_GLEAN_10016074), a helicase-encoding gene, were differentially expressed at the early and late time points.

### 3.5. Confirm Gene Expression Using Real-time Quantitative Reverse Transcription PCR

**Figure 5:**
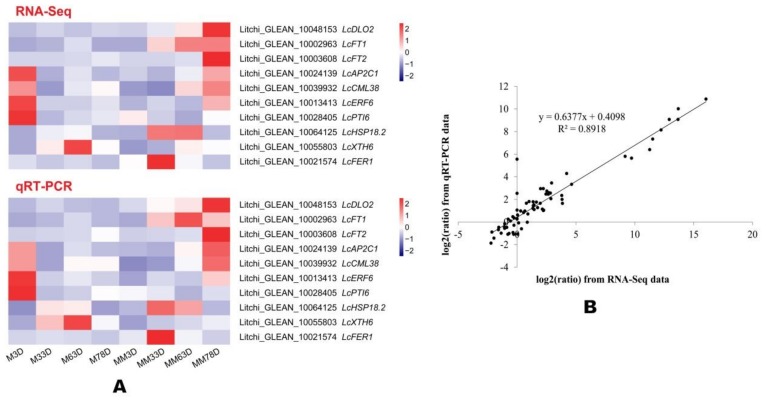
Heat map diagram of the tested gene expression levels revealed by quantitative real-time RT-PCR (qRT-PCR) and RNA-sequencing (RNA-Seq) (**A**), and the correlation between qRT-PCR and RNA-Seq of the 10 tested genes (**B**). ‘Nuomici’ litchi trees were transferred to a growth chamber at 18 °C/13 °C (day/night temperature, 12 h day and 12 h night) as medium-temperature treatment. The trees were sprayed with (MV as the medium-temperature plus MV treatment (MM) and water as the medium-temperature control (M). M3D, M33D, M63D, and M78D indicate 3 d, 33 d, 63 d, 78 of water treatment, respectively. MM3D, MM33D, MM63D, and MM78D respectively, indicate 3 d, 33 d, 63 d, 78 d of MV treatment under medium-temperature condition. Relative expression levels and FPKM values were normalized to Z-score.

To confirm the accuracy of the transcriptome analysis results, we randomly selected 10 genes for real-time quantitative reverse transcription-PCR (qRT-PCR) confirmation. As shown in Figure 5A, the expression profiles of the selected genes revealed by qRT-PCR data showed similar trends compared with those obtained from sequencing. We then analyzed the linear regression of the fold change of the gene expression ratios between RNA-Seq and qRT-PCR. As revealed in Figure 5B, the linear regression showed a positive correlation (R^2^ = 0.8918), suggesting that the transcriptome analysis by RNA-Seq is reliable.

### 3.6. Expression Levels of the Candidate Genes in Response to ROS and the ROS Scavenger

**Figure 6:**
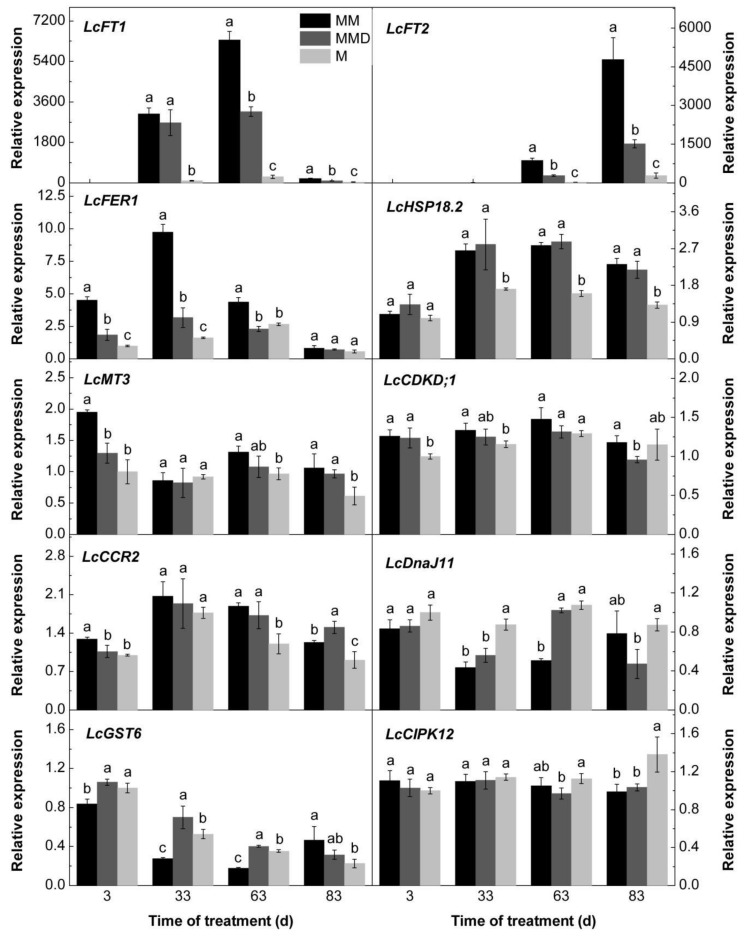
Relative expression profiles of the candidate genes in response to ROS. ‘Nuomici’ trees were transferred to a growth chamber at 18 °C/13 °C (day/night temperature, 12 h day and 12 h night) as medium-temperature treatment. The trees were sprayed with 40 µM MV as the medium-temperature plus MV treatment (MM), 2 mM N,N’-dimethylthiourea (DMTU) in combination with 40 µM MV as the medium-temperature plus DMTU and MV treatment (MMD), and water as the medium-temperature control (M). Data are the means of three replicates, and bars represent standard error (SE). Different letters indicate significant differences among treatments at a specific time point (P < 0.05, Duncan’s multiple range test).

To further confirm the effects of MV-generated ROS on the expression of the candidate genes, we selected 10 DEGs from the early and late ROS responsive genes, including *LcFT1*, *LcFT2*, *Ferritin 1* (*LcFER1*, Litchi_GLEAN_10021574), *18.2 kDa Heat Shock Protein* (*LcHSP18.2*, Litchi_GLEAN_10064125), *Metallothionein 3* (*LcMT3*, Litchi_GLEAN_10054139), *Cyclin-dependent kinase D1* (*LcCDKD;1*, Litchi_GLEAN_10002692), *Cold, circadian rhythm, and rna binding 2* (*LcCCR2*, Litchi_GLEAN_10035335), *Chaperone-domain superfamily protein dnaJ 11* (*LcDnaJ11*, Litchi_GLEAN_10044599), *Glutathione S-transferase 6* (*LcGST6*, Litchi_GLEAN_10048427), *CBL-interacting protein kinase 12* (*LcCIPK12*, Litchi_GLEAN_10039887) (Figure 6). We compared the gene expression levels of water-treated trees, ROS-treated trees, and ROS in combination with the ROS scavenger DMTU-treated trees. Relative expression of *LcFT1* showed that MV-generated ROS treatment increased its expression at 63 d and 83 d time points, but this effect was reduced by the ROS scavenger DMTU. Similar trends were found in the expression of *LcFT2* at 63 d and 83 d time points and that of *LcFER1* at 3 d, 33 d, 63 d time points, *LcMT3* at 3 d and 63 d time points, *LcCDKD;1* at 33 d time point, and *LcCCR2* at 3 d time point. At the 63 d time point, ROS decreased the relative expression of *LcDnaJ11*, but this reduction was repressed by the ROS scavenger DMTU. Similar trends were found in the expression of *LcGST6* at the 3 d, 33 d and 63 d time points. However, the expression of *LcHSP18.2* and *LcCIPK12* did not show any increased or decreased trends by ROS and the ROS plus DMTU treatments. The results showed that eight out of the 10 genes were further confirmed to be responsive to ROS treatment.

### 3.7. Co-Expression Network of the DEGs Expressed in Leaves

**Figure 7:**
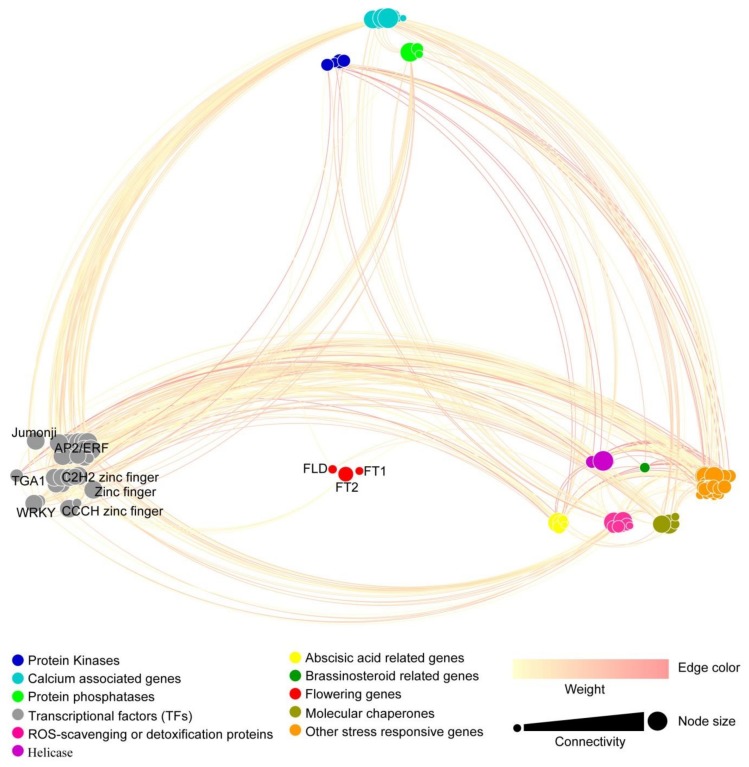
ROS-related co-expression network in litchi leaves. Ninety-three DEGs were selected among the 517 DEGs by using the weighted gene co-expression network analysis (WGCNA) R package, and a weight value > 0.15 was subjected as the threshold value. The connecting lines (edges) of color from light yellow to pink correspond to the weight value, and deeper colors represent higher values. The size of the node corresponds to the connectivity analyzed by NetworkAnalyzer of Cytoscape associated with the degrees (number of the edges) and weight value. Detailed information on the nodes and edges is shown in Table S3.

**Figure 8:**
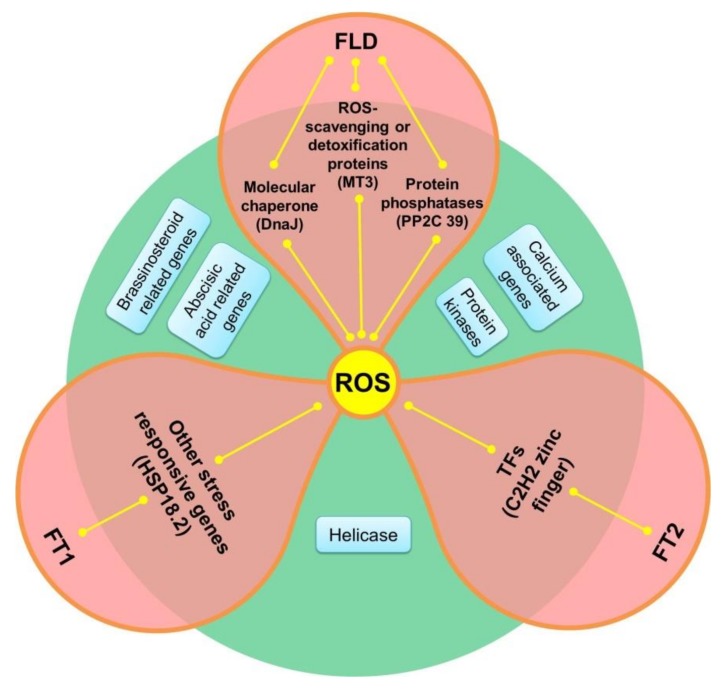
Co-expression network of the flowering-related genes showing detailed information of the three identified flowering-related genes from the ROS-related co-expression network. *LcFT2* is connected with four C2H2 zinc finger-type TF-encoding genes, *LcFT1* is related to *LcHSP18.2*, and *LcFLD* is associated with *LcDnaJ*, *LcMT3*, and *LcPP2C39.* The other five kinds of genes like protein kinases, helicase, calcium-, brassinosteroid- and abscisic acid-related genes had no direct interactions with the three flowering genes.

To analyze the gene linkage of the DEGs potentially involved in ROS regulation, we constructed a co-expression network based on weighted gene co-expression network analysis (WGCNA). Ninety-three DEGs were selected among the 517 DEGs. As shown in Figure 7 and Table S3, 11 kinds of genes were connected. *CIPK12*, *AP2C1*, *calcium-binding protein CML45* (Litchi_GLEAN_10022624), *ERF domain protein 9* (*ERF9,* Litchi_GLEAN_10053652), *microsomal glutathione* S-*transferase 3* (*MGST3,* Litchi_GLEAN_10034648), *PMH2*, *DnaJ11* (Litchi_GLEAN_10044599), nine-cis-*epoxycarotenoid dioxygenase 3* (*NCED3,* Litchi_GLEAN_10061007), *disease resistance family protein* (Litchi_GLEAN_10036444), and *brassinosteroid*-*insensitive 4* (Litchi_GLEAN_10040947, *BIN4*) encoding genes showed high connectivity in each of the 11 categories. The protein kinase genes *LcCIPK12* and *LcCDKD;1* showed a low degree (4 and 3) but high connectivity (15.5 and 12). Among the three flowering related genes, *LcFT2* had the highest connectivity, which was connected with four C2H2 zinc finger-type transcription factor (TF)-encoding genes. *LcFLD* was associated with *LcDnaJ*, *LcMT3*, and *LcPP2C39*. *LcFT1* was related to *LcHSP18.2* (Figure 7 and Figure 8).

## 4. Discussion

Stress is defined as a situation in which the vegetative growth of plants is suppressed [17]. Flowering is a transition from vegetative growth to reproductive growth under the premise of the suppression of vegetative growth. Hence, stress may play an important role in flowering. Many evergreen woody trees have to experience environmental stresses, such as chilling, drought, or oxidative stresses, to induce flowering [3]. Stress-induced flowering is an important method for plant survival. The stressed plants do not need to wait for the arrival of a season when photoperiodic conditions are suitable for flowering [17]. In our previous study, we showed that chilling stress caused ROS accumulation and induced flowering under relatively low temperature (15/8 °C, day/night) conditions [9]. The MV-generated ROS treatment could promote flowering under medium low-temperature (18/13 °C) conditions. We identified chilling- and ROS-responsive genes in SAMs from trees under chilling, medium chilling in the presence or absence of ROS, and non-chilling conditions and revealed an inner network of chilling- and ROS-promoted flowering in SAMs of litchi [11]. In Arabidopsis, rice, and cucurbit, the flowering signals, FT protein, or its analog have been shown to arise from leaves and then migrate to SAMs to initiate flowering [5,6,7,8]. 

In this study, we focused on the leaves from which flowering signals can be produced. ROS treatment and control at four time points (3 d, 33 d, 63 d, and 78 d) under medium-temperature conditions were carried out. We constructed 16 RNA-Seq libraries. After mapping to the litchi genome database, we found that the alignment rate of the 16 libraries was above 85%, indicating the excellent quality of the libraries. A total of 517 DEGs were obtained, among which 175 DEGs were from the 3 d time point, and 358 DEGs were from later time points. By selecting those related to ROS signaling, stress responsive and flowering-related genes, 48 out of 175 DEGs (27.27%) at 3 d of treatment, and 60 out of 358 DEGs (16.76%) at 33, 63, and 78 d of treatment were identified. Interestingly, more transcription factor-encoding genes were found at the 3 d time point, the early stage, while flowering-related genes were found at later time points. The results suggested that TFs might play roles in the early response of ROS. It might be that the early responsive TFs trigger floral induction activity and then initiate the expression of flowering related genes.

To further confirm the effects of ROS on the expression of the candidate genes, we used an ROS scavenger DMTU to trap ROS [18,19]. In accordance with our previous studies [9] and the present results of the ROS treatment, ROS promoted flowering, but this effect was reduced by DMTU. We then selected 10 DEGs from the RNA-Seq data set to further confirm the effects of MV-generated ROS on the expression of the candidate genes. We compared their expression patterns of the ROS, ROS plus DMTU, and control treatments. We found that 8 out of the 10 genes were responsive to ROS treatment at specific time points, suggesting that the reliability of the identified ROS-responsive genes from our RNA-Seq data is high. These eight genes were *LcFT1*, *LcFT2*, *LcFER1*, *LcMT3*, *LcCDKD;1*, *LcCCR2*, *LcDnaJ11* and *LcGST6*. The genes might be related to ROS-promoted flowering, for example, *LcFT1* and *LcFT2*.

In litchi, *LcFT*1 and *LcFT2* are proven to play critical roles in flowering, and overexpression of the two genes could promote flowering [20]. *LcFT1* is also involved in drought-enhanced flowering [21]. In the present study, the relative expression of *LcFT*1 and *LcFT2* was induced by ROS treatment, but the induction was trapped by the ROS scavenger DMTU, suggesting that they might play crucial roles in ROS-promoted flowering. 

In Arabidopsis, *FER1* plays an essential role in floral development and fertility. The ferritin-lacking triple mutant *fer1-3-4* shows that the loss of ferritins reduces growth and impairs flower development [22]. Our results showed that the relative expression of *LcFER1* was induced by ROS treatment, but the induction was repressed by the ROS scavenger DMTU, suggesting that it might be involved in ROS-promoted flowering in litchi. To date, we have not obtained any overexpression information regarding *LcFER1,* and further functional studies on *LcFER1* should be carried out. 

*LcCCR2* is the homolog of *AtGRP7*. In Arabidopsis, *AtGRP7* promotes flowering, its mutants and *AtGRP7i* plants are late flowering, and the transcript levels of *FLC* are affectced, which reveals the involvement of vernalization and autonomous pathways [23]. Interestingly, overexpression of *AtGRP7* in rice (*Oryza sativa*) under drought stress conditions showed higher recovery rates, and grain yields compared to the control wild-type plants [24]. Metallothioneins (MTs) are intracellular cysteine-rich, metal-binding proteins that function in metal homeostasis, ROS scavenging, oxidative damage limiting, and enhanced tolerance of many kinds of abiotic stress in plants [25]. *AtMT2a* and *AtMT3* overexpressed in *Vicia faba* guard cells could increase resistance to cadmium [26]. According to Xue et al. [27], *GhMT3a* could function as an effective ROS scavenger in cotton, transgenic tobacco, and yeast, and its expression was induced by various abiotic stresses through ROS signaling. In rice, OsMT-3a plays a crucial role in salinity and heavy metal tolerance via ROS scavenging [28]. In our study, the relative expression of *LcCCR2* and *LcMT2* was induced by ROS treatment at the early stage of floral induction, but the induction was trapped by the ROS scavenger DMTU, suggesting that they might be involved in ROS-promoted flowering at an early stage of floral induction. At present, we do not know whether overexpression of *LcCCR2* or *LcMT2* can promote flowering. Further functional studies on *LcMT2* should be carried out. Similarly, other candidate genes, such as *LcCDKD;1*, *LcCCR2*, *LcDnaJ11* and *LcGST6*, should be subjected to functional studies.

For further studies of the interaction between the ROS responsive genes, we established a co-expression network and divided those 11 groups into four categories according to the classification method from You et al. and Sudawan et al. [15,16]. Interestingly, the protein kinase genes *LcCIPK12* and *LcCDKD;1* showed low degree but high connectivity, mostly because of the higher weight value compared to protein phosphatases, calcium-associated genes, and the other genes. *LcFT2* among the flowering genes had the highest connectivity, which was connected with four C2H2 zinc finger-type TF-encoding genes. These results suggested that the protein kinase genes *LcCIPK12* and *LcCDKD;1*, and the flowering-related gene *LcFT2* may play central roles in ROS-promoted flowering in litchi.

## 5. Conclusions

We constructed 16 RNA-Seq libraries of ROS treatment and control samples at four time points (3 d, 33 d, 63 d, and 78 d) under medium-temperature conditions, and 517 DEGs were obtained. Among those DEGs, plant hormone biosynthesis and signal induction genes, ROS-specific transcription factors, such as AP2/ERF and WRKY genes, stress response genes, and flowering-related genes *FT1* and *FT2*, were significantly enriched. A confirmatory experiment in which potted trees were uniformly sprayed with MV, N,N’-dimethylthiourea (DMTU, ROS scavenger) plus MV, and water at medium-temperature was carried out. The results showed that the MV-generated ROS promoted flowering and changed the expression of related genes, but these effects were repressed by DMTU. 

Our previous studies have shown that the ROS promoted flowering and decreased the chilling requirement for flowering at the ecological and anatomical levels and obtained an inner network of chilling- and ROS-promoted flowering in the apical meristem of litchi. The studies revealed that ROS could promote flowering and partly bypass chilling requirements for litchi flowering, suggesting a possible litchi flowering promotion method for addressing climate change and global warming. 

## Figures and Tables

**Table 1:** Effects of medium-temperature, reactive oxygen species (ROS)-generator, and scavenger treatments on litchi trees.

Treatments	Percentage of Flowering Trees (%)	Length of Panicle/cm	Width of Panicle/cm	Max Spike Nodes per Panicle	No. of Flowers per Panicle
MM	80	12.61 ± 1.97	5.68 ± 1.79	11.35 ± 1.10 *	749.48 ± 180.41 *
MMD	80	10.99 ± 1.19	2.62 ± 0.53	7.20 ± 1.06	202.32 ± 73.29
M	0	-	-	-	-

Each of the five trees was sprayed with 40 µM MV as the medium-temperature plus MV treatment (MM), 2 mM DMTU in combination with 40 µM MV as the medium-temperature plus DMTU and MV treatment (MMD), and water as the medium-temperature control (M). Values of length, width, max spike nodes, and number of flowers are means ± SE from five replicate trees. Each value in one replicate was calculated from five panicles. Significant differences (*P* < 0.05, Student’s *t*-test) between the MM and the MMD are indicated by asterisks.

**Table 2:** Data quality and alignment analysis.

Sample	Raw Reads	Clean Reads	Clean Reads/Raw Reads (%)	Alignment Rate (%)
M3D-1	7838012	6906535	88.115902	85.87
M3D-2	7885494	7206931	91.394794	87.22
M33D-1	7768367	7505505	96.616252	87.9
M33D-2	6978483	6833631	97.924305	88.35
M63D-1	10799631	10360566	95.934444	88.45
M63D-2	9667348	9354050	96.759215	88.85
M78D-1	8278464	7879395	95.179432	85.84
M78D-2	9337570	8769018	93.911135	88.83
MM3D-1	7864489	7701724	97.93038	87.84
MM3D-2	11179790	10754878	96.199285	86.8
MM33D-1	8149987	7930713	97.309517	86.79
MM33D-2	5167534	4951442	95.818276	89.31
MM63D-1	9581811	8874327	92.616385	87.28
MM63D-2	12149900	11554502	95.099565	85.2
MM78D-1	9088200	8815661	97.001177	89.61
MM78D-2	11964902	10606541	88.64712	86.02

‘Nuomici’ trees were transferred to a growth chamber at 18°C/13°C (day/night temperature, 12 h day and 12 h night) as medium-temperature treatment. The trees were sprayed with methyl (MV as the medium-temperature plus MV treatment (MM) and water as the medium-temperature control (M). Samples at the first column of the table orderly mean 3 d (M3D), 33 d (M33D), 63 d (M63D), 78 d (M78D) of water treatment, and 3 d (MM3D), 33 d (MM33D), 63 d (MM63D), 78 d (MM78D) of MV treatment under medium-temperature condition. The Arabic numbers 1 and 2 represent the replications.

## Supplementary Materials

The following are available online at https://www.mdpi.com/2073-4425/11/3/324/s1, Figure S1: Heat map showing the correlation of the 16 RNA-Seq libraries, Table S1: Primer sequences of the reference gene and candidate genes for qRT-PCR, Table S2: Differentially expressed unigenes screened showing the FPKM value and the annotation of the 517 DEGs, Table S3: The detail information of the node and edge of the co-expression network.
